# Targeted proteomics-determined multi-biomarker profiles developed classifier for prognosis and immunotherapy responses of advanced cervical cancer

**DOI:** 10.3389/fimmu.2024.1391524

**Published:** 2024-05-21

**Authors:** Jin Li, Xu Zhang, Liuke Yang, Youwei Zhu, Rongrong Gao, Tiancheng Zhang, Xuwen Chen, Jun Fu, Gaoyang He, Huijuan Shi, Shenjie Peng, XiaoHua Wu

**Affiliations:** ^1^ Department of Gynecologic Oncology, Fudan University Shanghai Cancer Center, Fudan University, Shanghai, China; ^2^ Department of Oncology, Shanghai Medical College, Fudan University, Shanghai, China; ^3^ NHC Key Lab of Reproduction Regulation, Shanghai Engineering Research Center of Reproductive Health Drug and Devices, Shanghai Institute for Biomedical and Pharmaceutical Technologies, Shanghai, China; ^4^ Shanghai-MOST Key Laboratory of Health and Disease Genomics, NHC Key Lab of Reproduction Regulation, Shanghai Institute for Biomedical and Pharmaceutical Technologies, Shanghai, China; ^5^ College of Plant Protection, Nanjing Agricultural University, Nanjing, China; ^6^ Clinical Center of Bio-Therapy at Zhongshan Hospital & Institutes of Biomedical Sciences, Shanghai Public Health Clinical Center, Fudan University, Shanghai, China; ^7^ Clinical Center for Biotherapy at Zhongshan Hospital, Fudan University, Shanghai, China; ^8^ Shanghai Kelin Clinical Bioinformatics Institute, Shanghai, China; ^9^ LC-Bio Technology Co., Ltd, Hangzhou, China; ^10^ Shanghai Medical College of Fudan University, Fudan University, Shanghai, China

**Keywords:** cervical cancer, biomarker, proteomics, prognosis, immunotherapy response

## Abstract

**Background:**

Cervical cancer (CC) poses a global health challenge, with a particularly poor prognosis in cases of recurrence, metastasis, or advanced stages. A single biomarker is inadequate to predict CC prognosis or identify CC patients likely to benefit from immunotherapy, presumably owing to tumor complexity and heterogeneity.

**Methods:**

Using advanced Olink proteomics, we analyzed 92 oncology-related proteins in plasma from CC patients receiving immunotherapy, based upon the comparison of protein expression levels of pre-therapy with those of therapy-Cycle 6 in the partial response (PR) group and progressive disease (PD) group, respectively.

**Results:**

55 proteins were identified to exhibit differential expression trends across pre-therapy and post-therapy in both PR and PD groups. Enriched GO terms and KEGG pathways were associated with vital oncological and immunological processes. A logistic regression model, using 5 proteins (ITGB5, TGF-α, TLR3, WIF-1, and ERBB3) with highest AUC values, demonstrated good predictive performance for prognosis of CC patients undergoing immunotherapy and showed potential across different cancer types. The effectiveness of these proteins in prognosis prediction was further validated using TCGA-CESC datasets. A negative correlation and previously unidentified roles of WIF-1 in CC immunotherapy was also first determined.

**Conclusion:**

Our findings reveal multi-biomarker profiles effectively predicting CC prognosis and identifying patients benefitting most from immunotherapy, especially for those with limited treatment options and traditionally poor prognosis, paving the way for personalized immunotherapeutic treatments and improved clinical strategies.

## Introduction

Cervical cancer (CC) is the fourth leading cause of cancer-related deaths among women worldwide (1). Increasing rates of human papilloma- virus vaccination in recent years has led to a decline in the incidence and mortality of CC, nevertheless, there remains a significant burden especially among vulnerable populations without access to healthcare, particularly in developing countries and regions (2). As of 2020, China recorded approximately 110,000 newly diagnosed cases and 59,000 mortalities due to CC, positioning it as the second largest CC-burdened country in the world (3, 4). Future projections are even more concerning, with an estimated 75,000 CC deaths anticipated by 2040, marking an increase of 26.3% from 2020 (3, 4). Moreover, the prognosis, despite significant advances in prophylactic vaccinations, early detection and conventional treatments like chemotherapy and radiotherapy, still remains bleak for those with recurrent, metastatic, or advanced CC, where the 5-year survival rate hovers around 17.0% (1, 4).

While platinum-containing combination chemotherapy plus angiogenesis-targeted therapeutic agents (e.g. bevacizumab) is the preferred first-line treatment for non-radically treatable recurrent or metastatic CC (5) , disease progression is, sadly, often inevitable. The emergence of promising novel therapeutic options such as immunotherapy, particularly immune checkpoint inhibitors (ICIs), offers hope. The U.S. Food and Drug Administration (FDA) has approved the use of pembrolizumab in conjunction with chemotherapy, optionally combined with bevacizumab, as the first-line therapeutic approach for patients with persistent, recurrent, or metastatic CC presenting PD-L1 expression (CPS ≥1) (6). This approval was based upon the results of KEYNOTE-826 clinical trial in 2021 (7), underscoring the significant efficacy potential of ICIs for treating CC. Despite these advances marking significant strides in CC treatment, the inherent complexity of cancer precludes a uniform benefit across all patients, highlighting the imperative need to further refine our understanding and approaches. Presently, we’re devoid of robust tools to efficiently categorize CC patients who would optimally respond to ICIs immunotherapeutic regimen, nor can we predict ensuing immune responses and discern the best therapeutic amalgamations. The dire need for reliable and actionable prognostic and predictive immunological biomarkers cannot be overstated.

Protein biomarkers have been central to disease prediction, diagnosis as well as prevention. Advances in high-throughput proteomics, particularly through technologies like Olink, enable the simultaneous quantification of a multitude of proteins. Olink technology requiring small sample volumes crucial for sparse clinical samples, not only offers multiple assay panels targeted toward a variety of disease processes but also can capture a wide range of proteins across the entire dynamic range, exceeding 10 logarithmic scales (8). Additionally, Olink proteomics has been reported to demonstrate high reproducibility and stability in protein detection in plasma samples (9). In the present study, Olink proteomics is utilized to characterize protein profile differences between CC patients exhibiting partial response (PR) and progressive disease (PD) to ICIs immunotherapy, aiming to unravel the differences in immunological responses and identify potential biomarkers associated with these divergent treatment outcomes.

## Results

### Characteristics of the cervical cancer patients

**Figure 1:**
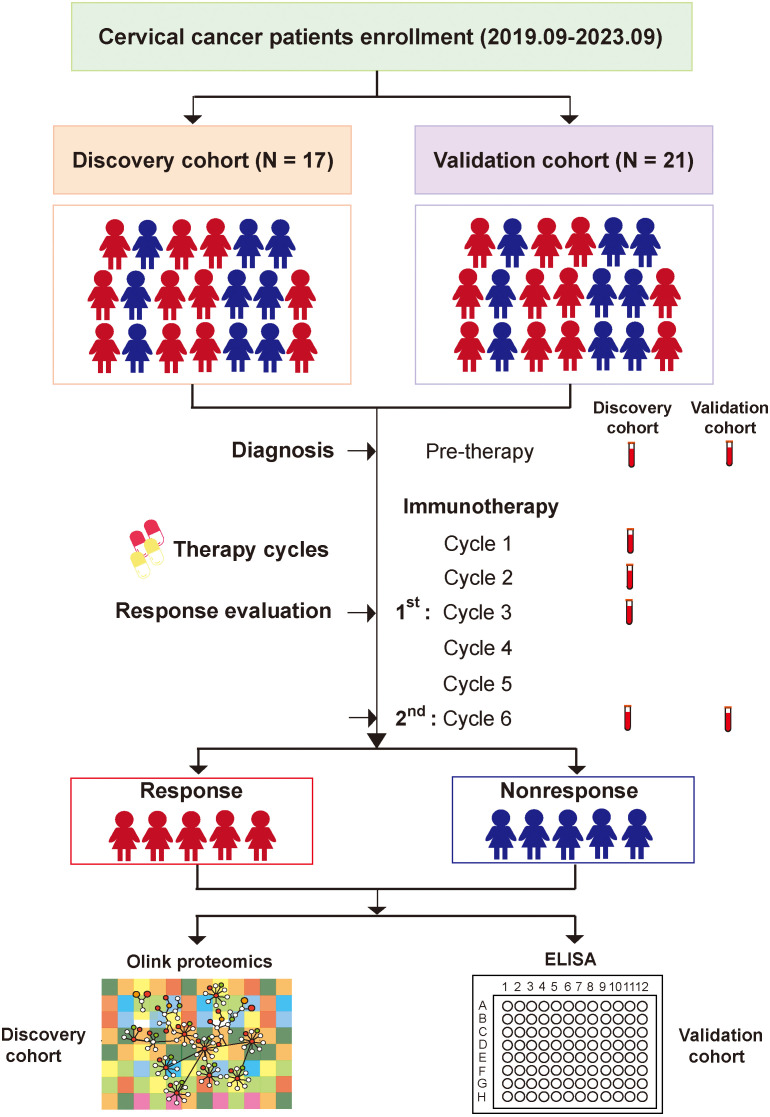
Flow chat of experimental design.

Clinical characteristics of 38 CC patients in discovery and validation cohorts are shown in **Table 1**. According to treatment outcome, these patients were divided into PR (partial response) and PD (progressive disease) groups (**Tables 2**, **3**, **Figure 1**). No significant differences were observed in age, pathological type, ECOG (Eastern Cooperative Oncology Group) score, stage, HPV infection status, PD-L1 expression (combined positive score, CPS), line of therapy, objective response rate (ORR), tumor extent at enrollment, and treatment before enrollment between the discovery cohort and validation cohort (**Table 1**). Within the discovery cohort, there were no significant differences in age, pathological type, ECOG score, stage, HPV infection status, CPS, line of therapy, ORR, tumor extent at enrollment, and treatment before enrollment between the PR and PD groups (**Table 2**). Similarly, within the validation cohort, between the PR and PD groups, age, pathological type, ECOG score, stage, HPV infection status, CPS, line of therapy, ORR, tumor extent at enrollment, and treatment before enrollment showed no significant differences (**Table 3**).

**Table 1:** Baseline characteristics of 38 cervical cancer patients who received immunotherapy in the discovery cohort and validation cohort.

Characteristic^*^	All n = 38	Discovery Cohort n = 17	Validation Cohort n = 21	P value^§^
Age (years)				
Mean ± SD	51.83 ± 6.71	53.49 ± 5.11	50.32 ± 7.38	0.53
Pathological type n (%)				
squamous	35 (92.11%)	16 (94.12%)	19 (90.48%)	0.061
adenocarcinoma	3 (7.89%)	1 (5.88%)	2 (9.52%)	
ECOG score n (%)				
0	35 (92.11%)	17 (100%)	18 (85.71%)	0.72
1	3 (7.89%)	0 (0.00%)	3 (14.29%)	
2	0 (0.00%)	0 (0.00%)	0 (0.00%)	
3	0 (0.00%)	0 (0.00%)	0 (0.00%)	
4	0 (0.00%)	0 (0.00%)	0 (0.00%)	
5	0 (0.00%)	0 (0.00%)	0 (0.00%)	
Stage n (%)				
IB1	6 (15.79%)	3 (17.65%)	3 (14.29%)	0.71
IB2	3 (7.89%)	1 (5.88%)	2 (9.52%)	
IIA	3 (7.89%)	2 (11.76%)	1 (4.76%)	
IIA1	5 (13.16%)	2 (11.76%)	3 (14.29%)	
IIB	2 (5.26%)	0 (0.00%)	2 (9.52%)	
IIIB	3 (7.89%)	1 (5.88%)	2 (9.52%)	
IIIC1p	7 (18.43)	5 (29.42%)	2 (9.52%)	
IVB	9 (23.69%)	3 (17.65%)	6 (28.58%)	
HPV infection status n (%)				
positive	22 (57.89%)	10 (58.82%)	12 (57.14%)	0.26
negative	3 (7.89%)	3 (17.65%)	0 (0.00%)	
unknown	13 (34.11%)	4 (23.53%)	9 (42.86%)	
PD-L1 expression (CPS) n (%)				
≥1	19 (50.00%)	9 (52.95%)	10 (47.62%)	0.063
<1	6 (15.79%)	2 (11.76%)	4 (19.05%)	
unknown	13 (34.21%)	6 (35.29%)	7 (33.33%)	
Line of therapy n (%)				
1	20 (52.63%)	9 (52.94%)	11 (52.38%)	0.34
2	15 (39.48%)	7 (41.18%)	8 (38.10%)	
≥3	3 (7.89%)	1 (5.89%)	2 (9.52%)	
Type of anti-PD-1/PD-L1 therapy n (%)				
Monotherapy	19 (50.00%)	9 (52.95%)	10 (47.62%)	3.69E-6
Combination therapy				
Chemotherapy	10 (26.32%)	6 (35.29%)	4 (19.05%)	
Anti-VEGF	7 (18.42%)	2 (11.76%)	5 (23.81%)	
Anti-CTLA-4	2 (5.26%)	0 (0.00%)	2 (9.52%)	
ORR n (%)				
CR/PR	23 (60.53%)	11 (64.71%)	12 (57.14%)	0.89
SD/PD	15 (39.47%)	6 (35.29%)	9 (42.86%)	
Tumor extent at enrollment n (%)				
locoregionally recurrent or persistent with metastasis	19 (50.00%)	9 (52.94%)	10 (47.62%)	0.29
metastasis without locoregional recurrence or persistence	10 (26.32%)	5 (29.42%)	5 (23.81%)	
no locoregionally recurrent, with metastasis	3 (7.89%)	1 (5.88%)	2 (9.52%)	
metastasis	2 (5.26%)	1 (5.88%)	1 (4.76%)	
left common iliac paravascular lymph node is locoregional recurrence and mediastinal lymph node was metastasis disease	4 (10.53%)	1 (5.88%)	3 (14.29%)	
Treatment before enrollment n (%)				
operation	5 (13.16%)	2 (11.76%)	3 (14.29%)	0.39
chemoradiotherapy	6 (15.79%)	2 (11.76%)	4 (19.05%)	
operation and chemoradiotherapy	8 (21.05%)	5 (29.42%)	3 (14.29%)	
operation and radiotherapy	13 (34.21%)	6 (35.30%)	7 (33.32%)	
no	6 (15.79%)	2 (11.76%)	4 (19.05%)	

ECOG, Eastern Cooperative Oncology Group; HPV, human papillomavirus; CPS, combined positive score; ORR, objective response rate; CR, complete response; PR, partial response; SD, stable disease; PD, progressive disease.

^*^Percentage indicates the proportion of patients with a specific clinical, pathologic, or molecular characteristic among all patients.

^§^To compare characteristics between subgroups, we used the χ^2^ test for categorical variables and Mann–Whitney U test for non-normally distributed continuous variables.

**Table 2:** Baseline characteristics of 17 cervical cancer patients who received immunotherapy in discovery cohort.

Characteristic^*^	Discovery Cohort n = 17	PR group n = 11	PD group n = 6	P value^§^
Age (years)				
Mean ± SD	53.49 ± 5.11	51.26 ± 3.49	55.53 ± 9.57	0.50
Pathological type n (%)				
squamous	16 (94.12%)	11 (100%)	5 (83.33%)	0.12
adenocarcinoma	1 (5.88%)	0 (0.00%)	1 (16.67%)	
ECOG Score n (%)				
0	17 (100%)	11(100%)	6 (100%)	0.80
1	0 (0.00%)	0 (0.00%)	0 (0.00%)	
2	0 (0.00%)	0 (0.00%)	0 (0.00%)	
3	0 (0.00%)	0 (0.00%)	0 (0.00%)	
4	0 (0.00%)	0 (0.00%)	0 (0.00%)	
5	0 (0.00%)	0 (0.00%)	0 (0.00%)	
Stage n (%)				
IB1	3 (17.65%)	2 (18.17%)	1 (16.67%)	0.87
IB2	1 (5.88%)	1 (9.10%)	0 (0.00%)	
IIA	2 (11.76%)	1 (9.10%)	1 (16.67%)	
IIA1	2 (11.76%)	1 (9.10%)	1 (16.67%)	
IIB	0 (0.00%)	0 (0.00%)	0 (0.00%)	
IIIB	1 (5.88%)	1 (9.10%)	0 (0.00%)	
IIIC1p	5 (29.42%)	3 (27.26%)	2 (33.32%)	
IVB	3 (17.65%)	2 (18.17%)	1 (16.67%)	
HPV infection status n (%)				
positive	10 (58.82%)	6 (54.55%)	4 (66.67%)	0.31
negative	3 (17.65%)	1 (9.09%)	2 (33.33%)	
unknown	4 (23.53%)	4 (36.36%)	0 (0.00%)	
PD-L1 expression (CPS) n (%)				
≥1	9 (52.95%)	4 (36.36%)	5 (83.33%)	0.066
<1	2 (11.76%)	1 (9.09%)	1 (16.67%)	
unknown	6 (35.29%)	6 (54.55%)	0 (0.00%)	
Line of therapy n (%)				
1	9 (52.94%)	6 (54.55%)	3 (50%)	0.66
2	7 (41.18%)	5 (45.45%)	2 (33.33%)	
≥3	1 (5.89%)	0 (0.00%)	1 (16.67%)	
Type of anti-PD-1/PD-L1 therapy n (%)				
Monotherapy	9 (52.95%)	7 (63.64%)	2 (33.33%)	1.17E-7
Combination therapy				
Chemotherapy	6 (35.29%)	4 (36.36%)	2 (33.33%)	
Anti-VEGF	2 (11.76%)	0 (0.00%)	2 (33.33%)	
Anti-CTLA-4	0 (0.00%)	0 (0.00%)	0 (0.00%)	
Tumor extent at enrollment n (%)				
locoregionally recurrent or persistent with metastasis	9 (52.94%)	6 (54.55%)	3 (50.00%)	0.49
metastasis without locoregional recurrence or persistence	5 (29.42%)	4 (36.35%)	1 (16.67%)	
no locoregionally recurrent, with metastasis	1 (5.88%)	1 (9.10%)	0 (0.00%)	
metastasis	1 (5.88%)	0 (0.00%)	1 (16.67%)	
left common iliac paravascular lymph node is locoregional recurrence and mediastinal lymph node was metastasis disease	1 (5.88%)	0 (0.00%)	1 (16.67%)	
Treatment before enrollment n (%)				
operation	2 (11.76%)	2 (18.18%)	0	0.63
chemoradiotherapy	2 (11.76%)	2 (18.18%)	0	
operation and chemoradiotherapy	5 (29.42%)	3 (27.27%)	2 (33.33%)	
operation and radiotherapy	6 (35.30%)	3 (27.27%)	3 (50.00%)	
no	2 (11.76%)	1 (9.10%)	1 (16.67%)	

ECOG, Eastern Cooperative Oncology Group; HPV, human papillomavirus; CPS, combined positive score; ORR, objective response rate; CR, complete response; PR, partial response; SD, stable disease; PD, progressive disease.

^*^Percentage indicates the proportion of patients with a specific clinical, pathologic, or molecular characteristic among all patients.

^§^To compare characteristics between subgroups, we used the χ^2^ test for categorical variables and Mann–Whitney U test for non-normally distributed continuous variables.

**Table 3:** Baseline characteristics of 21 cervical cancer patients who received immunotherapy in validation cohort.

Characteristic^*^	Validation Cohort n = 21	PR group n = 12	PD group n = 9	P value^§^
Age (years)				
Mean ± SD	50.32 ± 7.38	52.71 ± 6.77	48.33 ± 3.24	0.67
Pathological type n (%)				
squamous	19 (90.48%)	12 (100%)	7 (77.78%)	0.058
adenocarcinoma	2 (9.52%)	0	2 (22.22%)	
ECOG Score n (%)				
0	18 (85.71%)	10 (83.33%)	8 (88.89%)	0.77
1	3 (14.29%)	2 (16.67%)	1 (11.11%)	
2	0	0	0	
3	0	0	0	
4	0	0	0	
5	0	0	0	
Stage n (%)				
IB1	3 (14.29%)	2 (16.67%)	1 (11.11%)	0.56
IB2	2 (9.52%)	1 (8.33%)	1 (11.11%)	
IIA	1 (4.76%)	1 (8.33%)	0	
IIA1	3 (14.29%)	2 (16.67%)	1 (11.11%)	
IIB	2 (9.52%)	1 (8.33%)	1 (11.11%)	
IIIB	2 (9.52%)	2 (16.67%)	0	
IIIC1p	2 (9.52%)	1 (8.33%)	1 (11.11%)	
IVB	6 (28.58%)	2 (16.67%)	4 (44.45%)	
HPV infection status n (%)				
positive	12 (57.14%)	6 (50.00%)	6 (66.67%)	0.45
negative	0	0	0	
unknown	9 (42.86%)	6 (50.00%)	3 (33.33%)	
PD-L1 expression (CPS) n (%)				
≥1	10 (47.62%)	3 (25.00%)	7 (77.78%)	0.071
<1	4 (19.05%)	2 (16.67%)	2 (22.22%)	
unknown	7 (33.33%)	7 (58.33%)	0	
Line of therapy n (%)				
1	11 (52.38%)	8 (66.67%)	3 (33.33%)	0.54
2	8 (38.10%)	3 (25.00%)	5 (55.56%)	
≥3	2 (9.52%)	1 (8.33%)	1 (11.11%)	
Type of anti-PD-1/PD-L1 therapy n (%)				
Monotherapy	10 (47.62%)	7 (58.33%)	3 (33.33%)	2.55E-4
Combination therapy				
Chemotherapy	4 (19.05%)	1 (8.33%)	3 (33.33%)	
Anti-VEGF	5 (23.81%)	3 (25.00%)	2 (22.22%)	
Anti-CTLA-4	2 (9.52%)	1 (8.33%)	1 (11.11%)	
Tumor extent at enrollment n (%)				
locoregionally recurrent or persistent with metastasis	10 (47.62%)	7 (58.33%)	3 (33.33%)	0.33
metastasis without locoregional recurrence or persistence	5 (23.81%)	2 (16.67%)	3 (33.33%)	
no locoregionally recurrent, with metastasis	2 (9.52%)	1 (8.33%)	1 (11.11%)	
metastasis	1 (4.76%)	0	1 (11.11%)	
left common iliac paravascular lymph node is locoregional recurrence and mediastinal lymph node was metastasis disease	3 (14.29%)	2 (16.67%)	1 (11.11%)	
Treatment before enrollment n (%)				
operation	3 (14.29%)	1 (8.33%)	2 (22.22%)	0.27
chemoradiotherapy	4 (19.05%)	2 (16.67%)	2 (22.22%)	
operation and chemoradiotherapy	3 (14.29%)	2 (16.67%)	1 (11.11%)	
operation and radiotherapy	7 (33.32%)	3 (25.00%)	4 (44.45%)	
no	4 (19.05%)	4 (33.33%)	0	

ECOG, Eastern Cooperative Oncology Group; HPV, human papillomavirus; CPS, combined positive score; ORR, objective response rate; CR, complete response; PR, partial response; SD, stable disease; PD, progressive disease.

^*^Percentage indicates the proportion of patients with a specific clinical, pathologic, or molecular characteristic among all patients.

^§^To compare characteristics between subgroups, we used the χ^2^ test for categorical variables and Mann–Whitney U test for non-normally distributed continuous variables.

### Olink oncology-related biomarker analysis

**Figure 2:**
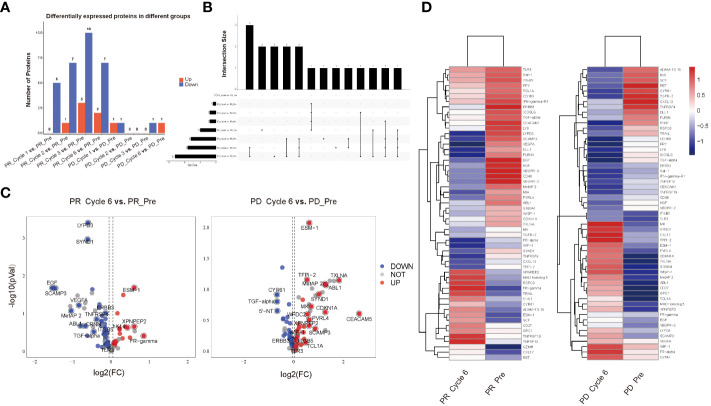
All differentially expressed oncology-related proteins in PR and PD groups. **(A)** Bar graph of differentially expressed oncology-related proteins among different groups. **(B)** UpSet diagram of differentially expressed oncology-related proteins shared among different groups. **(C)** Volcano plots illustrating the comparison of expression levels of 92 oncology-related proteins of pre-therapy and those of therapy-Cycle 6 in the PR group (left) and PD group (right), with blue dots representing proteins that were downregulated, red dots representing proteins that were upregulated, and gray dots representing proteins with no significant change in expression. **(D)** Heatmap of 55 proteins showing differential expression trends shared by PR and PD groups. PR_Pre, pre-therapy in PR group; PR_Cycle 1, therapy-cycle 1 in PR group; PR_Cycle 2, therapy-cycle 2 in PR group; PR_Cycle 3, therapy-cycle 3 in PR group; PR_Cycle 6, therapy-cycle 6 in PR group; PD_Pre, pre-therapy in PD group; PD_Cycle 1, therapy-cycle 1 in PD group; PD_Cycle 2, therapy-cycle 2 in PD group; PD_Cycle 3, therapy-cycle 3 in PD group; PD_Cycle 6, therapy-cycle 6 in PD group.

We evaluated and compared expression levels of 92 oncology-related proteins (**Supplementary Table 1**) in PR and PD groups. A total of 9 and 2 significantly differentially expressed oncology-related proteins were identified through comparing protein expression of pre-therapy and that of therapy-Cycle 6 in PR and PD groups, respectively, of which 2 were upregulated and 7 were downregulated in PR group, and 1 upregulated and 1 downregulated proteins were observed in PD group (**Figures 2A, B**). Due to the small number of proteins with expression differences reaching a statistically significant level, when comparing protein expression levels between pre-therapy and therapy-Cycle 6, proteins with an estimate value of ≥0.05 or ≤-0.05 and a mean Normalized Protein eXpression (NPX) value of ≥3.5 were selected as candidate molecules. Subsequently, within PR and PD groups, 63 and 61 proteins were respectively identified which, although not achieving statistical significance, demonstrated trends towards differential expression (**Figure 2C**). In addition, 55 proteins exhibiting differential expression trends were shared between these two groups (**Supplementary Table 2**) and **Figure 2D** showed the heatmap of these overlapping proteins.

### Analysis of biomarkers related to oncology and immunology

**Figure 3:**
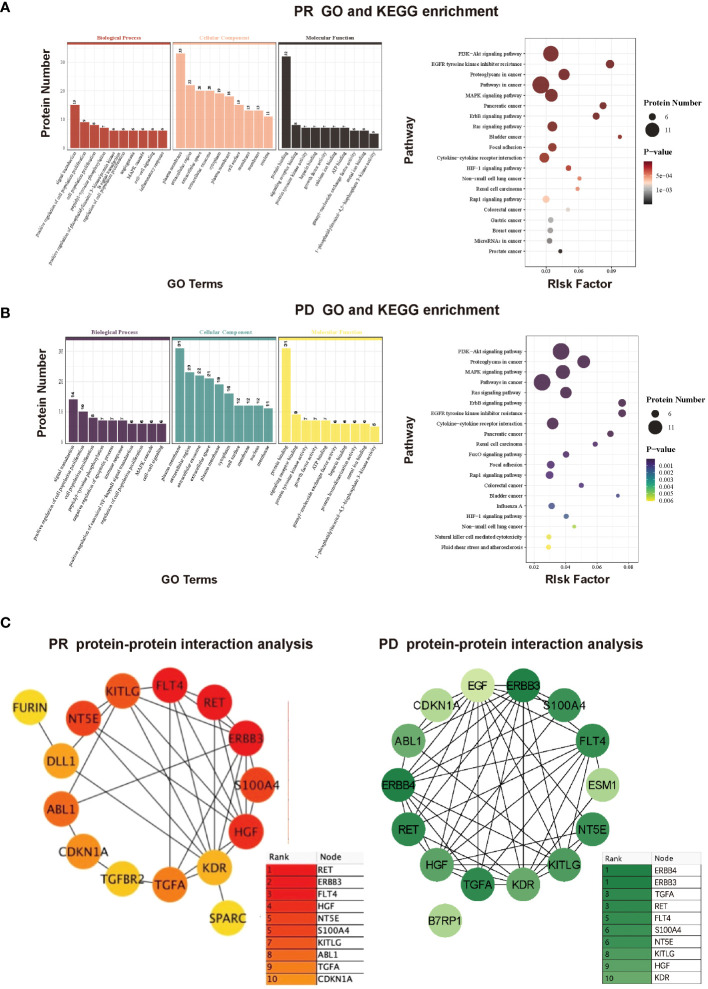
**(A)** GO and KEGG enrichment analyses of proteins with differential expression trends between pre-therapy and therapy-Cycle 6 in PR group. **(B)** GO and KEGG enrichment analyses of proteins with differential expression trends between pre-therapy and therapy-Cycle 6 in PD group. **(C)** Protein-protein interaction (PPI) network analysis of proteins with differential expression trends between pre-therapy and therapy-Cycle 6 in PR (left) and PD (right) groups.

To further investigate potential functions of the above-mentioned proteins, GO and KEGG enrichment analyses were conducted based on various proteins with differential expression trends identified by comparing protein expression of pre-therapy with that of therapy-Cycle 6 in PR and PD groups. Within the PR group, the results indicated that these proteins were enriched in several GO terms such as regulation of cell population proliferation, angiogenesis, and cell-cell signaling as well as pathways related to oncology and immunology, like PI3K-Akt signaling pathway, pathways in cancer, MAPK signaling pathway, ErbB signaling pathway, and focal adhesion (**Figure 3A**). What’s more, enrichment analysis in PD group showed that the proteins were enriched in multiple GO terms and KEGG pathways, including regulation of cell population proliferation, immune response, and cell-cell signaling, as well as a number of oncology and immunology-related pathways such as PI3K-Akt signaling pathway, MAPK signaling pathway, pathways in cancer, ErbB signaling pathway, focal adhesion, and natural killer cell mediated cytotoxicity (**Figure 3B**).

Furthermore, a protein-protein interaction (PPI) network was also characterized, in which the top 15 hub proteins, selected based on the highest PPI scores through the DMNC algorithm, are predominantly associated with immune responses or signal transduction (10, 11) . Moreover, compared to the PR group, the PPI score and ranking of proto-oncogene tyrosine-protein kinase receptor Ret (Ret) in PD group were notably reduced (0.7683 PR; 0.6706 PD), erb-b2 receptor tyrosine kinase 3 (ERBB3) remained unchanged (0.6957 PR; 0.6957 PD), and the PPI score and ranking of transforming growth factor alpha (TGF-α) were both elevated (0.5967 PR; 0.6706 PD), suggesting that the interactive effects of ERBB3 and TGF-α were significant within the protein interaction networks of PD group, and TGF-α may have a greater impact in PD (**Figure 3C**).

### Principal component analysis and predictive model for prognosis of cervical cancer patients receiving immunotherapy

**Figure 4:**
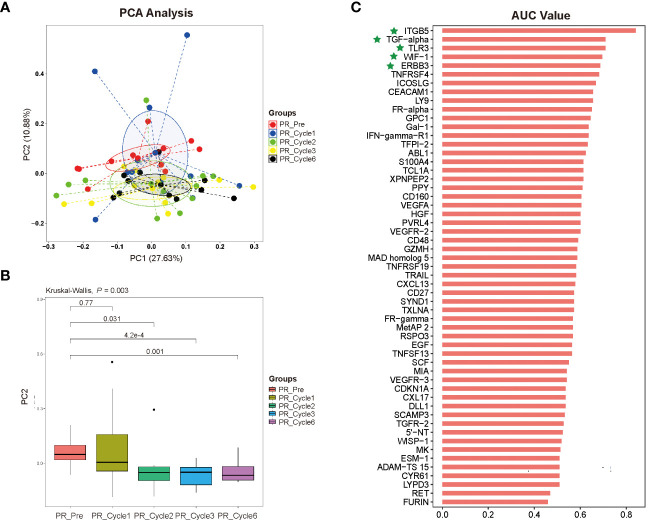
**(A)** Distribution of dimension 1 (PC 1) and dimension 2 (PC 2) based upon principal component analysis (PCA) of 92 proteins in different therapy cycles of PR group. **(B)** Pairwise comparison with principal component 2 (PC2), in comparison with pre-therapy of PR group. **(C)** Bar plot of 92 proteins for predicting prognosis of ICIs immunotherapy-treated patients with persistent, recurrent, or metastatic CC, representing an area of curve (AUC) value for each protein, with proteins ITGB5, TGF-α, TLR3, WIF-1, and ERBB3 highlighted by green stars.

The results from principal component analysis of PR patients were shown in **Figure 4A**, in which the numbers on the axes represented the variation captured by each principal component. Levels of 92 plasma proteins were explained 38.51% by the first two principal components (PC1 27.63%, PC2 10.88%, respectively), and pre-therapy group was separated from therapy-Cycle 2, therapy-Cycle 3 and therapy-Cycle 6 groups, respectively, with PC2. Thus, the PC2 distribution across different groups was illustrated in **Figure 4B**, and Kruskal-Wallis analysis found a significant difference among these groups (P = 0.003) and pairwise comparison showed that pre-therapy group, in PR patients, was significantly distinct from therapy-Cycle 2 group, therapy-Cycle 3 group as well as therapy-Cycle 6 group (P = 0.031, P = 4.2×10–^4^, P = 0.001). In contrast, no obvious separation or significant difference was observed in PD patients (data not shown).

**Figure 5:**
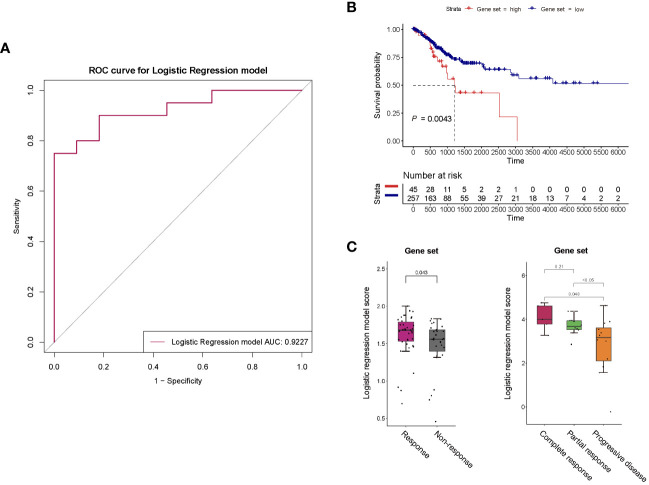
**(A)** ROC curve for the binary logistic regression model established with proteins ITGB5, TGF-α, TLR3, WIF-1, and ERBB3. The predictive power of this predictive model was assessed using area under the curve (AUC). **(B)** Survival analysis of the 5-gene set in general patients with cervical cancer. **(C)** Response of the 5-gene set to anti-PD-1 monotherapy and anti-PD-1/anti-CTLA-4 combined therapy of melanoma.

To identify potential protein biomarkers of prognosis prediction of ICIs immunotherapy-treated patients with persistent, recurrent, or metastatic CC, the area under the curve (AUC) values of 92 proteins were shown in **Figure 4C**. We established a predictive model using binary logistic regression analysis, in which the AUC value of each of 55 overlapping proteins was calculated, followed by the selection of top 5 proteins with highest AUC values (ITGB5, TGF-α, TLR3, WIF-1, and ERBB3; **Figure 4C**; **Supplementary Table 4**) for binary logistic regression model analysis of which receiver operating characteristics (ROC) results were used for evaluating model’s predictive performance. In ROC curve, the AUC value used for the assessment of protein profile-associated predictive power was 0.9227 (**Figure 5A**), proving the model’s effectiveness and indicating a good performance in predicting prognosis of immunotherapy-treated CC patients. In other words, these above-mentioned top 5 proteins could serve as potential biomarkers for prognostic prediction of CC patients receiving immunotherapy treatment.

### Survival analysis in general patients and response to immune checkpoint therapy

For survival analysis in general patients, we utilized RNA-seq data matrices and clinical information for the TCGA cancer datasets of cervical squamous cell carcinoma and endocervical adenocarcinoma (CESC) obtained from UCSC-xena. We specifically selected the primary solid tumor (code 01) of TCGA-CESC, which was the majority of the samples in TCGA-CESC dataset (302 out of 312). Next, a scoring model was established according to the binary logistic regression model above, which was a combination of expression levels of the 5 aforementioned proteins (ITGB5, TGF-α, TLR3, WIF-1, and ERBB3) (collectively referred to as “5-gene get” in this study). If the protein was more highly expressed in PR group than PD group in our cohort study, a positive weight was given, conversely, a negative weight was assigned for a lower expression in PR group. That meant, our model calculation formula applied positive coefficient weights (value is positive 1/+ 1) to genes ITGB5, TGF-α, TLR3 and ERBB3, and a negative coefficient weight (value is minus 1/- 1) to gene WIF-1. The final risk score for each CC patient was then obtained through a weighted average. Based on the risk score, these TCGA-CESC samples were stratified into 45 high-risk (with higher score) and 257 low-risk groups (with lower score). The results showed that a negative association between 5-gene-set scores and survival of general CC patients was statistically significant (P = 0.0043) (**Figure 5B**).

Next, we further analyzed responses to ICIs immunotherapy in CC based on the 5-gene-set with a thorough search of published CC immunotherapy studies containing all these 5 genes but did not find any. Therefore, we utilized melanoma immunotherapy studies, in which clinical and gene expression data were obtained from published studies on melanoma patients receiving anti-PD-1 monotherapy, anti-CTLA-4 monotherapy or anti-PD-1/anti-CTLA-4 combined therapy (12–15). The sample number was based upon the selection of patients with primary tumors (patients without primary tumors were not included), thus, the number of patients analyzed was less than that in published studies. Except for the PR (partial response), CR (complete response), PD (progressive disease) and SD (stable disease) groups shown in figures, PR and CR were classified as the response group, as well as SD and PD as the non-response group within the rest figures. The related information was collected based on RECIST or response records from published datasets, and the scoring method for 5-gene set here was similar with that in survival analysis. Higher score of the 5-gene set was observed in responders to both anti-PD-1 monotherapy and anti-PD-1/anti-CTLA-4 combined therapy of melanoma (P = 0.043, P = 0.048, P < 0.05) (**Figure 5C**), suggesting that our predictive model may be capable of predicting effects of ICIs immunotherapy with targets such as PD-1 and CTLA-4 for different cancer types.

These results further proving the effectiveness of these 5 proteins in prognostic prediction of CC patients suggested that CC patients with higher 5-gene-set scores tend to have poorer prognosis, nevertheless, better responses to ICIs immunotherapy with targets like PD-1 and CTLA-4 were also demonstrated by them, which strongly indicated that these patients were more suitable for ICIs immunotherapy.

### ELISA validation and model performance

**Figure 6:**
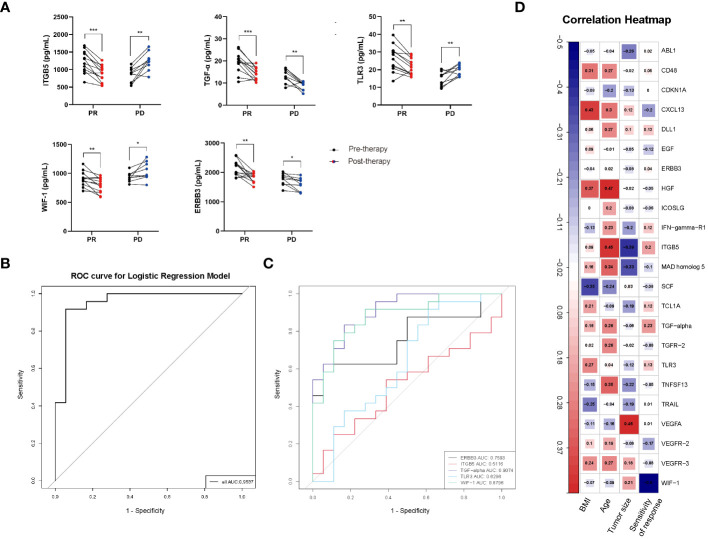
**(A)** ELISA validation of proteins ITGB5, TGF-α, TLR3, WIF-1 and ERBB3 in CC patients’ plasma. PR: partial response; PD: progressive disease; P values calculated via paired t test *: P < 0.05, **: P < 0.01, ***: P < 0.001, ns: not significant. **(B)** ROC curve of predictive model containing ITGB5, TGF-α, TLR3, WIF-1 and ERBB3. **(C)** AUC values of ITGB5, TGF-α, TLR3, WIF-1 and ERBB3. **(D)** Correlation heatmap between 55 overlapping proteins and clinical features of CC patients. Red, positively related; blue, negatively related.

According to the ELISA results, in PR group of CC patients’ plasma, expression levels of ITGB5, TGF-α, TLR3, WIF-1, and ERBB3 were significantly higher at pre-therapy time point compared with that at post-therapy time point (therapy-Cycle 6) (P < 0.001, P < 0.001, P < 0.01, P < 0.01, P < 0.01; **Figure 6A**), which was consistent with the trend observed in the Olink data. In contrast, in PD group of CC patients’ plasma, expression levels of TGF-α and ERBB3 were significantly higher at the pre-therapy time point (P < 0.01, P < 0.05; **Figure 6A**), while expression levels of ITGB5, TLR3, and WIF-1 were significantly lower at the pre-therapy time point in comparison with those at post-therapy time point (therapy-Cycle 6) (P < 0.01, P < 0.01, P < 0.05; **Figure 6A**).

Based on the ELISA results, the effectiveness of these 5 protein markers in distinguishing between good and poor prognosis was evaluated. The ROC curve analysis yielded AUC values of 0.5116 for ITGB5, 0.9074 for TGF-α, 0.6296 for TLR3, 0.8796 for WIF-1 as well as 0.7593 for ERBB3, respectively (**Figure 6C**). What’s more, the AUC value of predictive model containing ITGB5, TGF-α, TLR3, WIF-1, and ERBB3 was 0.9537 (**Figure 6B**), further proving established model’s effectiveness and good performance in predicting prognosis of CC patients receiving immunotherapy treatment.

In addition, in the analysis of correlations between the 55 overlapping proteins and the clinical features of CC patients, an opposite trend was observed in the associations of proteins ITGB5, TGF-α, TLR3, WIF-1, and ERBB3 with the clinical feature of sensitivity of immunotherapy response. Increased expression levels of ITGB5, TGF-α, TLR3, and ERBB3 were observed to be proportional to CC patients’ sensitivity of immunotherapy response, while higher level of WIF-1 was inversely associated with the response sensitivity in CC patients under immunotherapy (**Figure 6D**).

## Discussion

In advanced cervical cancer, especially recurrent or metastatic cases, therapy options after first-line treatment failure are limited. The U.S. FDA approval of pembrolizumab, in combination with chemotherapy, for PD-L1-positive CC highlights great therapeutic potential of ICIs (16–18). However, challenges like minimal efficacy, adverse events, therapy resistance, and disease progression persist in ICIs immunotherapy, including monotherapy (1, 19, 20) and combination therapies (19, 21, 22). Identifying patients who will benefit from ICIs, predicting treatment outcomes, and selecting appropriate strategies are of importance in clinical practice. In the present study, we identify potential protein biomarkers related to prognosis and immunological responses to ICIs immunotherapy for advanced CC using Olink proteomics.

In our study, we utilized a comparatively large, nationally representative sample (n = 38) of CC patients receiving immunotherapy, and comprehensively explored the plasma proteomic changes associated with responses to ICIs immunotherapy, expanding upon previous researches within this field (23, 24). A total of 63 proteins with differential expression trends were identified in the PR group, and 61 in the PD group, by comparing protein expression levels of pre-therapy group and that of therapy-Cycle 6 group, among which 55 proteins were common to both groups (**Figure 2**). These proteins were involved in key oncology and immunology pathways, including PI3K-Akt, MAPK, and NK cell-mediated cytotoxicity signaling (**Figure 3**). Upon calculating the AUC values of each previously mentioned protein, we developed different predictive models based on binary logistic regression analysis, which involved selecting the top 10 proteins according to their AUC values, ranked in descending order (**Supplementary Table 3**). Notably, the model with the top 5 proteins (ITGB5, TGF-α, TLR3, WIF-1, and ERBB3) (**Supplementary Table 4**) was found to be highly effective, showing the highest AUC value of 0.9227 and 0.9537 in the discovery and validation cohorts, respectively (**Figure 5A**, **Figure 6B**), indicating a strong predictive performance in prognosis of CC patients undergoing immunotherapy. Survival analysis using TCGA-CESC datasets showed a significant negative association between the logistic regression model score of 5-gene set (genes ITGB5, TGF-α, TLR3, WIF-1, ERBB3) and survival in general CC patients (**Figure 5B**). Given the limited research on CC immunotherapy involving all 5 genes, we turned to studies on melanoma for insights. We found that higher model scores of the 5-gene set were associated with better responses to anti-PD-1 and anti-CTLA-4 therapies (**Figure 5C**). This suggested that the model may be effective in predicting responses to ICIs immunotherapy across different cancers. Moreover, it indicated that CC patients with higher 5-gene-set scores, despite potentially poorer prognoses, may exhibit better responses to ICIs immunotherapy with targets like PD-1 and CTLA-4. Consequently, this model had the potential to facilitate the identification of cancer patients likely to respond well to ICIs immunotherapy before treatment.

The five proteins ITGB5, TGF-α, TLR3, WIF-1, and ERBB3, of which TGF-α and ERBB3 were PPI hub proteins (**Figure 3C**), play distinct yet interconnected roles in CC. ITGB5, associated with focal adhesion, immune suppression, and signaling pathways critical in cancer progression and poor survival outcomes in various cancers (25–28), is less studied in CC but is reported to activate the focal adhesion pathway, inducing cisplatin resistance in CC cells (29) . TGF-α, through its interaction with EGFR, regulates cell proliferation, differentiation, and migration, potentially forming immunosuppressive environments in CC and influencing immunotherapy effectiveness (30–32). TLR3, a crucial pattern recognition receptor in immune responses against HPV and cervical intra-epithelial neoplasia (33–36), enhances anti-tumor immunity (37, 38) but can also inadvertently promote tumor growth through promoting pro-inflammatory cytokine production (39, 40). WIF-1, a Wnt antagonist, is implicated in inhibiting metastasis and tumorigenesis in multiple cancers (41, 42) and is often epigenetically silenced in CC, affecting apoptosis and cancer growth (43, 44). ERBB3 influences PI3K/AKT/mTOR pathway activation and epithelial−mesenchymal transition (EMT) in CC (45–47), contributing to immune evasion and tumor development, and may interact with immune checkpoints to facilitate tumor escape (48–54).

Among these five proteins, higher expression levels of ITGB5 and TGF-α were proportional to the clinical feature of immunotherapy sensitivity, while WIF-1 was inversely associated with this feature (**Figure 6D**). Moreover, WIF-1 expression levels were observed to decrease in PR group and increase in PD group at therapy-Cycle 6 (**Figure 6A**), differing from previous findings of its anti-tumor effects in CC via the Wnt signaling pathway (55, 56). This might be attributed to factors such as tumor heterogeneity, interactions within TME (changes like immune infiltration within TME might affect WIF-1 expression), selective pressure induced by immunotherapy (ineffective immunotherapy might allow survival of treatment-resistant tumor cells, which might exhibit higher WIF-1 expression as a survival mechanism in PD patients), or the direct/indirect effects of immunotherapeutic drugs. A negative correlation of WIF-1 in CC immunotherapy was first determined in this study, uncovering previously unidentified roles and establishing its association with the prognosis of persistent, recurrent, or metastatic CC under immunotherapy treatment.

Overall, our research presents several contributory elements in the field of CC immunotherapy. Firstly, we conducted a comprehensive study involving a relatively large sample size, screening for protein biomarkers related to CC immunotherapy, which is crucial for enhancing our understanding and improving treatment outcomes. Secondly, our findings suggested that 5 specific proteins (ITGB5, TGF-α, TLR3, WIF-1, and ERBB3) could serve as potential biomarkers for prognostic prediction in immunotherapy-treated patients with persistent, recurrent, or metastatic CC and the identification of CC patients who likely would benefit from ICIs immunotherapy-containing treatment strategies. Furthermore, a notable discovery in this study was marked by our identification of the WIF-1 molecule, which was previously less known in its roles. Our findings uncovered a negative correlation between WIF-1 expression and the effectiveness of CC immunotherapy, alongside a connection with the prognosis of persistent, recurrent, or metastatic CC under such therapeutic interventions. In addition, the utilization of the Olink proteomics showing protein measurement-related high sensitivity, high specificity and high reproducibility (2, 3) underpin the robustness and reliability of our findings in the realm of CC immunotherapy research.

However, there are some limitations in the present study, such as the research being primarily conducted within a Chinese population, which may limit the applicability of our findings to other ethnic or racial groups. Additionally, future studies with larger sample sizes in diverse populations are necessary to confirm and extend our results, particularly in understanding the roles of specific proteins in CC. Our future research will be dedicated to the in-depth mechanistic exploration of WIF-1, especially its role in suppressing the TME within the context of CC immunotherapy.

## Materials and methods

### Patients

The studies involving human participants were reviewed and approved by Ethics Committee of Fudan University Shanghai Cancer Center (approval number: 050432-4-1805C). After exclusion of patients with concurrent autoimmune diseases, HIV, or syphilis, we obtained plasma samples from 38 patients, all of whom had histologically confirmed diagnoses of cervical cancer made between September 2019 and September 2023, prior to treatment (informed written consent from all participants was obtained prior to the research). During the treatment phase, which involved immunotherapy (alone or combination), an evaluation of the immunotherapeutic effects was conducted at an interval of every three treatment cycles, and CC patients’ plasma samples were collected at four time points: Cycle 1, Cycle 2, Cycle 3 (after 1^st^ response evaluation), and Cycle 6 (after 2^nd^ response evaluation). The collected plasma samples from the discovery cohort (n = 17) and validation cohort (n = 21) were respectively subjected to Olink proteomic analysis and ELISA tests (**Figure 1**).

### Plasma sample collection

Approximately 2 mL of peripheral venous blood was collected from each patient into EDTA tubes, followed by plasma extraction by centrifugation at 3000 rpm for 15 min, extracted plasma was frozen at −80°C for future research.

### Analysis of biomarkers related to oncology

Serum proteins from 17 CC patients were measured using Olink^®^ Target 96 Oncology II (v.7005) panel (Olink Proteomics AB, Uppsala, Sweden) according to manufacturer’s instructions. The Proximity Extension Assay (PEA) technology used for Olink protocol has been well described, enabling 92 analytes to be analyzed simultaneously, using 1 µL of each sample. Briefly, pairs of oligonucleotide-labeled antibody probes bound to their target protein, and the oligonucleotides would hybridize in a pair-wise manner when two probes were brought in close proximity. A proximity-dependent DNA polymerization event was caused by the addition of a DNA polymerase, generating a unique PCR target sequence. Resulting DNA sequence was subsequently detected and quantified using a microfluidic real-time PCR instrument (Signature Q100, LC-Bio Technology CO., Ltd., Hangzhou, China), followed by quality control (QC) and normalization of Ct-data using a set of internal and external controls. The final assay read-out was presented in Normalized Protein eXpression (NPX) values, which was an arbitrary unit on a log2-scale where one NPX difference equaled to a doubling of protein concentration. Four internal controls were designed to mimic and monitor different PEA steps, including two non-human proteins with matching antibody-probes as incubation/immuno controls, an IgG antibody with two attached matching probes as an extension control and a complete double-stranded amplicon as a detection control. These internal controls were introduced to all samples and to the external controls consisting of a triplicate of negative controls used for calculating limit of detection (LOD), as well as a triplicate of interplate controls (IPCs) containing 92 sets of antibodies with both matching probes for each assay attached to them used for normalization. For each sample and assay, NPX was calculated by following equations: 1. Ct(analyte) – Ct(extension control) = dCt(analyte) (to decrease technical variation) 2. dCt(analyte) – Ct(median IPC) = ddCt(analyte) (to improve inter plate variation) 3. Correction factor(analyte) – ddCt(analyte) = NPX(analyte) (for more intuitive data) (correction factor was a set variable unique for each assay and reagent lot). Data-related quality control was performed in two steps: First, the standard deviation (SD) for each of the incubation/immuno controls and detection control was calculated for each run, in which a run would only pass quality control if SD for each control was below 0.2; Second, each sample was quality controlled using incubation control 2 and detection control. The run median of each control was calculated, to which all samples within the run were compared. Samples falling more than +/- 0.3 NPX from the plate median regarding these two controls would fail quality control and receive a QC warning in data output file. All assay validation data such as detection limits, intra- and inter-assay precision data are available on manufacturer’s website (www.olink.com).

### Bioinformatics analysis

For Olink data, differentially expressed proteins were identified using OlinkAnalyze 3.4.1 package (olink_ttest) (Olink Statistical Analysis app - Olink) with a P-value cutoff of 0.05. Visualization of heat maps and volcano plots as well as enrichment analyses of Gene Ontology (GO) and Kyoto Encyclopedia of Genes and Genomes (KEGG) were all performed using OmicStudio tools (https://www.omicstudio.cn/tool). All significantly differentially expressed proteins were mapped to each term or pathway of GO or KEGG database in enrichment analyses, and the GO term or KEGG pathway which was significantly enriched in differentially expressed proteins compared to specific background was then identified using a hypergeometric test. Next, comparison of enrichment analysis results based on the background of all proteins and 92 proteins in Olink Oncology II; panel was performed. The protein-protein interaction (PPI) network of differentially expressed proteins was constructed and visualized using Cytoscape (version 3.9.1) (DMNC algorithm) (https://cytoscape.org/). In addition, the receiver operating characteristic (ROC) curves were created using ROCR package (4, 57).

### ELISA validation

Plasma samples from 21 CC patients were used to perform ELISA analysis using the ELISA kits for ITGB5 (SEC098Hu, Cloud-Clone Corp., USA), TGF-α (SEA123Hu, Cloud-Clone Corp., USA), TLR3 (SEB989Hu, Cloud-Clone Corp., USA), WIF-1 (SEL826Hu, Cloud-Clone Corp., USA), and ERBB3 (SEC187Hu, Cloud-Clone Corp., USA). Assays and analyses were conducted according to the manufacturer’s protocol.

### Statistical analysis

Data were presented as mean ± standard deviation (STD) or median (first and third quartiles) as appropriate. Statistical analyses were performed using IBM SPSS Statistics 25 and R software (version 4.1.3). P < 0.05 was considered statistically significant.

## Data availability statement

The original contributions presented in the study are included in the article/**Supplementary Materials**, further inquiries can be directed to the corresponding author/s.

## Ethics statement

The studies involving human participants were reviewed and approved by Ethics Committee of Fudan University Shanghai Cancer Center (approval number: 050432-4-1805C).The studies were conducted in accordance with the local legislation and institutional requirements. The participants provided their written informed consent to participate in this study.

## Author contributions

XZ: Funding acquisition, Investigation, Methodology, Supervision, Validation, Visualization, Writing – original draft, Writing – review & editing. JL: Validation, Writing – review & editing. LY: Methodology, Software, Visualization, Writing – review & editing. YZ: Investigation, Methodology, Software, Validation, Visualization, Writing – review & editing. RG: Software, Validation, Writing – review & editing. TZ: Supervision, Visualization, Writing – review & editing. XC: Supervision, Validation, Writing – review & editing. JF: Methodology, Software, Validation, Visualization, Writing – review & editing. GH: Methodology, Software, Validation, Visualization, Writing – review & editing. HS: Supervision, Writing – review & editing. SP: Software, Validation, Writing – review & editing. XW: Supervision, Writing – review & editing.
