# Corrigendum: Targeted proteomics-determined multi-biomarker profiles developed classifier for prognosis and immunotherapy responses of advanced cervical cancer

**DOI:** 10.3389/fimmu.2024.1437676

**Published:** 2024-07-02

**Authors:** Jin Li, Xu Zhang, Liuke Yang, Youwei Zhu, Rongrong Gao, Tiancheng Zhang, Xuwen Chen, Jun Fu, Gaoyang He, Huijuan Shi, Shenjie Peng, XiaoHua Wu

**Affiliations:** ^1^ Department of Gynecologic Oncology, Fudan University Shanghai Cancer Center, Fudan University, Shanghai, China; ^2^ Department of Oncology, Shanghai Medical College, Fudan University, Shanghai, China; ^3^ NHC Key Lab of Reproduction Regulation, Shanghai Engineering Research Center of Reproductive Health Drug and Devices, Shanghai Institute for Biomedical and Pharmaceutical Technologies, Shanghai, China; ^4^ Shanghai-MOST Key Laboratory of Health and Disease Genomics, NHC Key Lab of Reproduction Regulation, Shanghai Institute for Biomedical and Pharmaceutical Technologies, Shanghai, China; ^5^ College of plant protection, Nanjing Agricultural University, Nanjing, China; ^6^ Clinical Center of Bio-Therapy at Zhongshan Hospital & Institutes of Biomedical Sciences, Shanghai Public Health Clinical Center, Fudan University, Shanghai, China; ^7^ Clinical Center for Biotherapy at Zhongshan Hospital, Fudan University, Shanghai, China; ^8^ Shanghai Kelin Clinical Bioinformatics Institute, Shanghai, China; ^9^ LC-Bio Technology Co., Ltd, Hangzhou, China; ^10^ Fudan University, Shanghai, China

**Keywords:** cervical cancer, biomarker, proteomics, prognosis, immunotherapy response

In the published article, there was an error in affiliation 1, 2, 3, 4. Instead of “^1^NHC Key Lab of Reproduction Regulation, Shanghai Engineering Research Center of Reproductive Health Drug and Devices, Shanghai Institute for Biomedical and Pharmaceutical Technologies, Shanghai, China

^2^Shanghai-MOST Key Laboratory of Health and Disease Genomics, NHC Key Lab of Reproduction Regulation, Shanghai Institute for Biomedical and Pharmaceutical Technologies, Shanghai, China

^3^Department of Gynecologic Oncology, Fudan University Shanghai Cancer Center, Fudan University, Shanghai, China

^4^Department of Oncology, Shanghai Medical College, Fudan University, Shanghai 200032, China”, it should be “^1^Department of Gynecologic Oncology, Fudan University Shanghai Cancer Center, Fudan University, Shanghai, China

^2^Department of Oncology, Shanghai Medical College, Fudan University, Shanghai, China

^3^NHC Key Lab of Reproduction Regulation, Shanghai Engineering Research Center of Reproductive Health Drug and Devices, Shanghai Institute for Biomedical and Pharmaceutical Technologies, Shanghai, China

^4^Shanghai-MOST Key Laboratory of Health and Disease Genomics, NHC Key Lab of Reproduction Regulation, Shanghai Institute for Biomedical and Pharmaceutical Technologies, Shanghai 20037, China”.

In the published article, there was an error in the author list, and the first two authors appeared in the wrong order. The corrected author list appears below.

Jin Li^1,2,†^, Xu Zhang^3,4,†,^*, Liuke Yang^5^, Youwei Zhu^6^, Rongrong Gao^7^, Tiancheng Zhang^3,4^, Xuwen Chen^8^, Jun Fu^9^, Gaoyang He^9^, Huijuan Shi^3,4^, Shenjie Peng^10^, XiaoHua Wu^1,2,^*.

In the published article, there was an error of incorrect representation of the name of the ethics committee and errors in the choice between work email and personal email of co-correspondence author.

A correction has been made to Ethics statement and correspondence section. This sentence previously stated:

“The studies involving humans were approved by the ethics committee of Fudan University Cancer Hospital Shanghai, China.”

XiaoHua Wu xhwu2313@163.com

The corrected sentence appears below:

“The studies involving human participants were reviewed and approved by Ethics Committee of Fudan University Shanghai Cancer Center (approval number: 050432-4-1805C).”

XiaoHua Wu wu.xh@fudan.edu.cn

The authors apologize for these errors and state that this does not change the scientific conclusions of the article in any way. The original article has been updated.

