# Supplementary material for: Targeted proteomics-determined multi-biomarker profiles developed classifier for prognosis and immunotherapy responses of advanced cervical cancer
Source: Front Immunol. 2024 May 21;15:1391524. doi: 10.3389/fimmu.2024.1391524 (PMC11148239; doi:10.3389/fimmu.2024.1391524)
Supplement: Supplementary file 1 [file Table_1.docx]

**Supplemental Tables**

Supplemental Table S1. 92 oncology-related proteins.

| **OlinkID** | **Uniprot ID** | **Assay** | **Description** |
| --- | --- | --- | --- |
| OID00655 | P40222 | TXLNA | Alpha-taxilin |
| OID00656 | P15692 | VEGFA | Vascular endothelial growth factor A |
| OID00657 | P16870 | CPE | Carboxypeptidase E |
| OID00658 | Q9UKR3 | KLK13 | Kallikrein-13 |
| OID00659 | P13688 | CEACAM1 | Carcinoembryonic antigen-related cell adhesion molecule 1 |
| OID00660 | Q13421 | MSLN | Mesothelin |
| OID00661 | O75888 | TNFSF13 | Tumor necrosis factor ligand superfamily member 13 |
| OID00662 | P01133 | EGF | Pro-epidermal growth factor |
| OID00663 | O95407 | TNFRSF6B | Tumor necrosis factor receptor superfamily member 6B |
| OID00664 | P18827 | SYND1 | Syndecan-1 |
| OID00665 | P37173 | TGFR-2 | TGF-beta receptor type-2 |
| OID00666 | P05231 | IL6 | Interleukin-6 |
| OID00667 | P09326 | CD48 | CD48 antigen |
| OID00668 | O14828 | SCAMP3 | Secretory carrier-associated membrane protein 3 |
| OID00669 | Q9HBG7 | LY9 | T-lymphocyte surface antigen Ly-9 |
| OID00670 | P15260 | IFN-gamma-R1 | Interferon gamma receptor 1 |
| OID00671 | P06756 | ITGAV | Integrin alpha-V |
| OID00672 | P50591 | TRAIL | Tumor necrosis factor ligand superfamily member 10 |
| OID00673 | Q9UBX7 | hK11 | Kallikrein-11 |
| OID00674 | P35052 | GPC1 | Glypican-1 |
| OID00675 | P48307 | TFPI-2 | Tissue factor pathway inhibitor 2 |
| OID00676 | O60259 | hK8 | Kallikrein-8 |
| OID00677 | P35968 | VEGFR-2 | Vascular endothelial growth factor receptor 2 |
| OID00678 | O95274 | LYPD3 | Ly6/PLAUR domain-containing protein 3 |
| OID00679 | O00592 | PODXL | Podocalyxin |
| OID00680 | P26447 | S100A4 | S100 calcium binding protein A4 |
| OID00681 | P08069 | IGF1R | Insulin-like growth factor 1 receptor |
| OID00682 | P04626 | ERBB2 | erb-b2 receptor tyrosine kinase 2 |
| OID00683 | P21860 | ERBB3 | erb-b2 receptor tyrosine kinase 3 |
| OID00684 | P21583 | SCF | Kit ligand |
| OID00685 | P09486 | SPARC | SPARC |
| OID00686 | P20718 | GZMH | Granzyme H |
| OID00687 | P01135 | TGF-alpha | Protransforming growth factor alpha |
| OID00688 | P09958 | FURIN | Furin |
| OID00689 | O00622 | CYR61 | Protein CYR61 |
| OID00690 | Q9P0G3 | hK14 | Kallikrein-14 |
| OID00691 | Q13158 | FADD | FAS-associated death domain protein |
| OID00692 | P50579 | MetAP 2 | Methionine aminopeptidase 2 |
| OID00693 | Q96NY8 | PVRL4 | Nectin-4 |
| OID00694 | P48023 | FASLG | Tumor necrosis factor ligand superfamily member 6 |
| OID00695 | P29317 | EPHA2 | Ephrin type-A receptor 2 |
| OID00696 | P18084 | ITGB5 | Integrin beta-5 |
| OID00697 | P09382 | Gal-1 | Galectin-1 |
| OID00698 | Q9BYH1 | SEZ6L | Seizure 6-like protein |
| OID00700 | Q16790 | CAIX | Carbonic anhydrase 9 |
| OID00701 | Q16674 | MIA | Melanoma-derived growth regulatory protein |
| OID00702 | O60911 | CTSV | Cathepsin L2 |
| OID00703 | P26842 | CD27 | CD27 antigen |
| OID00704 | O43895 | XPNPEP2 | Xaa-Pro aminopeptidase 2 |
| OID00705 | Q15303 | ERBB4 | erb-b2 receptor tyrosine kinase 4 |
| OID00706 | P14210 | HGF | Hepatocyte growth factor |
| OID00707 | P78325 | ADAM 8 | Disintegrin and metalloproteinase domain-containing protein 8 |
| OID00708 | P21589 | 5'-NT | 5'-nucleotidase |
| OID00709 | P38936 | CDKN1A | Cyclin-dependent kinase inhibitor 1 |
| OID00710 | O00548 | DLL1 | Delta-like protein 1 |
| OID00711 | P21741 | MK | Midkine |
| OID00712 | P00519 | ABL1 | Tyrosine-protein kinase ABL1 |
| OID00713 | Q14512 | FGF-BP1 | Fibroblast growth factor-binding protein 1 |
| OID00714 | O15455 | TLR3 | Toll-like receptor 3 |
| OID00715 | P07948 | LYN | Tyrosine-protein kinase Lyn |
| OID00716 | P07949 | RET | Proto-oncogene tyrosine-protein kinase receptor Ret |
| OID00717 | P08670 | VIM | Vimentin |
| OID00718 | Q9NS68 | TNFRSF19 | Tumor necrosis factor receptor superfamily member 19 |
| OID00719 | Q9UBG3 | CRNN | Cornulin |
| OID00720 | P56279 | TCL1A | T-cell leukemia/lymphoma protein 1A |
| OID00721 | O95971 | CD160 | CD160 antigen |
| OID00722 | P43489 | TNFRSF4 | Tumor necrosis factor receptor superfamily member 4 |
| OID00723 | Q29983, Q29980 | MIC-A/B | - |
| OID00724 | O95388 | WISP-1 | WNT1-inducible-signaling pathway protein 1 |
| OID00725 | Q6UXB2 | CXL17 | C-X-C motif chemokine 17 |
| OID00726 | P01298 | PPY | Pancreatic prohormone |
| OID00727 | P31949 | S100A11 | Protein S100-A11 |
| OID00728 | P15514 | AREG | Amphiregulin |
| OID00729 | Q9NQ30 | ESM-1 | Endothelial cell-specific molecule 1 |
| OID00730 | Q9UJ71 | CD207 | C-type lectin domain family 4 member K |
| OID00731 | O75144 | ICOSLG | ICOS ligand OS=Homo sapiens |
| OID00732 | Q14508 | WFDC2 | WAP four-disulfide core domain protein 2 |
| OID00733 | O43927 | CXCL13 | C-X-C motif chemokine 13 |
| OID00734 | Q99717 | MAD homolog 5 | Mothers against decapentaplegic homolog 5 |
| OID00735 | Q8TE58 | ADAM-TS 15 | A disintegrin and metalloproteinase with thrombospondin motifs 15 |
| OID00736 | P32970 | CD70 | CD70 antigen |
| OID00737 | Q9BXY4 | RSPO3 | R-spondin-3 |
| OID00738 | P41439 | FR-gamma | Folate receptor gamma |
| OID00739 | P06731 | CEACAM5 | Carcinoembryonic antigen-related cell adhesion molecule 5 |
| OID00740 | P35916 | VEGFR-3 | Vascular endothelial growth factor receptor 3 |
| OID00741 | Q8WXI7 | MUC-16 | Mucin-16 |
| OID00742 | Q9Y5W5 | WIF-1 | Wnt inhibitory factor 1 |
| OID00743 | P10144 | GZMB | Granzyme B |
| OID00744 | Q6BAA4 | FCRLB | Fc receptor-like B |
| OID00745 | P04083 | ANXA1 | Annexin A1 |
| OID00746 | P15328 | FR-alpha | Folate receptor alpha |
| OID00749 | Q14956 | GPNMB | Transmembrane glycoprotein NMB |

Supplemental Table S2. 55 overlapping proteins with differential expression trends.

| **OlinkID** | **Uniprot ID** | **Assay** | **Description** |
| --- | --- | --- | --- |
| OID00655 | P40222 | TXLNA | Alpha-taxilin |
| OID00656 | P15692 | VEGFA | Vascular endothelial growth factor A |
| OID00659 | P13688 | CEACAM1 | Carcinoembryonic antigen-related cell adhesion molecule 1 |
| OID00661 | O75888 | TNFSF13 | Tumor necrosis factor ligand superfamily member 13 |
| OID00662 | P01133 | EGF | Pro-epidermal growth factor |
| OID00664 | P18827 | SYND1 | Syndecan-1 |
| OID00665 | P37173 | TGFR-2 | TGF-beta receptor type-2 |
| OID00735 | Q8TE58 | ADAM-TS 15 | A disintegrin and metalloproteinase with thrombospondin motifs 15 |
| OID00667 | P09326 | CD48 | CD48 antigen |
| OID00668 | O14828 | SCAMP3 | Secretory carrier-associated membrane protein 3 |
| OID00672 | P50591 | TRAIL | Tumor necrosis factor ligand superfamily member 10 |
| OID00675 | P48307 | TFPI-2 | Tissue factor pathway inhibitor 2 |
| OID00746 | P15328 | FR-alpha | Folate receptor alpha |
| OID00677 | P35968 | VEGFR-2 | Vascular endothelial growth factor receptor 2 |
| OID00678 | O95274 | LYPD3 | Ly6/PLAUR domain-containing protein 3 |
| OID00680 | P26447 | S100A4 | Protein S100-A4 |
| OID00683 | P21860 | ERBB3 | Receptor tyrosine-protein kinase erbB-3 |
| OID00684 | P21583 | SCF | Kit ligand |
| OID00697 | P09382 | Gal-1 | Galectin-1 |
| OID00686 | P20718 | GZMH | Granzyme H |
| OID00687 | P01135 | TGF-alpha | Protransforming growth factor alpha |
| OID00688 | P09958 | FURIN | Furin |
| OID00689 | O00622 | CYR61 | Protein CYR61 |
| OID00674 | P35052 | GPC1 | Glypican-1 |
| OID00692 | P50579 | MetAP 2 | Methionine aminopeptidase 2 |
| OID00693 | Q96NY8 | PVRL4 | Nectin-4 |
| OID00731 | O75144 | ICOSLG | ICOS ligand OS=Homo sapiens |
| OID00696 | P18084 | ITGB5 | Integrin beta-5 |
| OID00670 | P15260 | IFN-gamma-R1 | Interferon gamma receptor 1 |
| OID00701 | Q16674 | MIA | Melanoma-derived growth regulatory protein |
| OID00703 | P26842 | CD27 | CD27 antigen |
| OID00704 | O43895 | XPNPEP2 | Xaa-Pro aminopeptidase 2 |
| OID00706 | P14210 | HGF | Hepatocyte growth factor |
| OID00669 | Q9HBG7 | LY9 | T-lymphocyte surface antigen Ly-9 |
| OID00708 | P21589 | 5'-NT | 5'-nucleotidase |
| OID00709 | P38936 | CDKN1A | Cyclin-dependent kinase inhibitor 1 |
| OID00710 | O00548 | DLL1 | Delta-like protein 1 |
| OID00711 | P21741 | MK | Midkine |
| OID00712 | P00519 | ABL1 | Tyrosine-protein kinase ABL1 |
| OID00714 | O15455 | TLR3 | Toll-like receptor 3 |
| OID00734 | Q99717 | MAD homolog 5 | Mothers against decapentaplegic homolog 5 |
| OID00716 | P07949 | RET | Proto-oncogene tyrosine-protein kinase receptor Ret |
| OID00720 | P56279 | TCL1A | T-cell leukemia/lymphoma protein 1A |
| OID00721 | O95971 | CD160 | CD160 antigen |
| OID00722 | P43489 | TNFRSF4 | Tumor necrosis factor receptor superfamily member 4 |
| OID00737 | Q9BXY4 | RSPO3 | R-spondin-3 |
| OID00725 | Q6UXB2 | CXL17 | C-X-C motif chemokine 17 |
| OID00726 | P01298 | PPY | Pancreatic prohormone |
| OID00729 | Q9NQ30 | ESM-1 | Endothelial cell-specific molecule 1 |
| OID00718 | Q9NS68 | TNFRSF19 | Tumor necrosis factor receptor superfamily member 19 |
| OID00733 | O43927 | CXCL13 | C-X-C motif chemokine 13 |
| OID00738 | P41439 | FR-gamma | Folate receptor gamma |
| OID00740 | P35916 | VEGFR-3 | Vascular endothelial growth factor receptor 3 |
| OID00724 | O95388 | WISP-1 | WNT1-inducible-signaling pathway protein 1 |
| OID00742 | Q9Y5W5 | WIF-1 | Wnt inhibitory factor 1 |

Supplemental Table S3. AUC values of different protein combinations.

| **Protein combination** | **AUC value** |
| --- | --- |
| ITGB5, TGF-α | 0.5011 |
| ITGB5, TGF-α, TLR3 | 0.7122 |
| ITGB5, TGF-α, TLR3, WIF1 | 0.8919 |
| ITGB5, TGF-α, TLR3, WIF1, ERBB3 | 0.9227 |
| ITGB5, TGF-α, TLR3, WIF1, ERBB3, TNFRSF4 | 0.9098 |
| ITGB5, TGF-α, TLR3, WIF1, ERBB3, TNFRSF4, CEACAM1 | 0.8872 |
| ITGB5, TGF-α, TLR3, WIF1, ERBB3, TNFRSF4, CEACAM1, hK8 | 0.7965 |
| ITGB5, TGF-α, TLR3, WIF1, ERBB3, TNFRSF4, CEACAM1, hK8, FR-alpha | 0.7237 |

Supplemental Table S4. 5 overlapping proteins with the highest AUC values.

| **OlinkID** | **Uniprot ID** | **Assay** | **Description** |
| --- | --- | --- | --- |
| OID00683 | P21860 | ERBB3 | erb-b2 receptor tyrosine kinase 3 |
| OID00687 | P01135 | TGF-alpha | Transforming growth factor alpha |
| OID00696 | P18084 | ITGB5 | Integrin beta-5 |
| OID00714 | O15455 | TLR3 | Toll-like receptor 3 |
| OID00742 | Q9Y5W5 | WIF-1 | Wnt inhibitory factor 1 |
